# Mitigation of Nickel Toxicity and Growth Promotion in Sesame through the Application of a Bacterial Endophyte and Zeolite in Nickel Contaminated Soil

**DOI:** 10.3390/ijerph17238859

**Published:** 2020-11-28

**Authors:** Muhammad Naveed, Syeda Sosan Bukhari, Adnan Mustafa, Allah Ditta, Saud Alamri, Mohamed A. El-Esawi, Munazza Rafique, Sobia Ashraf, Manzer H. Siddiqui

**Affiliations:** 1 Institute of Soil and Environmental Sciences, University of Agriculture, Faisalabad 38040, Pakistan; sosansyed14@gmail.com (S.S.B.); sobiaashraf13@googlemail.com (S.A.); 2 National Engineering Laboratory for Improving Quality of Arable Land, Institute of Agricultural Resources and Regional Planning, Chinese Academy of Agricultural Sciences, Beijing 100081, China; adnanmustafa780@gmail.com; 3 Department of Environmental Sciences, Shaheed Benazir Bhutto University Sheringal, Upper Dir 18000, Pakistan; ad_abs@yahoo.com; 4 School of Biological Sciences, The University of Western Australia, Perth, WA 6009, Australia; 5 Department of Botany and Microbiology, College of Science, King Saud University, Riyadh 11451, Saudi Arabia; saudalmari@ksu.edu.sa (S.A.); manzerhs@yahoo.co.in (M.H.S.); 6 Botany Department, Faculty of Science, Tanta University, Tanta 31527, Egypt; mohamed.elesawi@science.tanta.edu.eg; 7 Soil Bacteriology Section, Ayub Agriculture Research Institute, Faisalabad 38000, Pakistan; munazzaaari@gmail.com

**Keywords:** Ni-contaminated soil, sesame, zeolite, *Caulobacter* sp. MN13, metal tolerant microbe, in situ immobilization, phytoremediation

## Abstract

Nickel (Ni) bioavailable fraction in the soil is of utmost importance because of its involvement in plant growth and environmental feedbacks. High concentrations of Ni in the soil environment, especially in the root zone, may retard plant growth that ultimately results in reduced plant biomass and yield. However, endophytic microorganisms have great potential to reduce the toxicity of Ni, especially when applied together with zeolite. The present research work was conducted to evaluate the potential effects of an endophytic bacterium *Caulobacter* sp. MN13 in combination with zeolite on the physiology, growth, quality, and yield of sesame plant under normal and Ni stressed soil conditions through possible reduction of Ni uptake. Surface sterilized sesame seeds were sown in pots filled with artificially Ni contaminated soil amended with zeolite. Results revealed that plant agronomic attributes such as shoot root dry weight, total number of pods, and 1000-grains weight were increased by 41, 45, 54, and 65%, respectively, over control treatment, with combined application of bacteria and zeolite in Ni contaminated soil. In comparison to control, the gaseous exchange parameters (CO_2_ assimilation rate, transpiration rate, stomatal- sub-stomatal conductance, chlorophyll content, and vapor pressure) were significantly enhanced by co-application of bacteria and zeolite ranging from 20 to 49% under Ni stress. Moreover, the combined utilization of bacteria and zeolite considerably improved water relations of sesame plant, in terms of relative water content (RWC) and relative membrane permeability (RMP) along with improvement in biochemical components (protein, ash, crude fiber, fat), and micronutrients in normal as well as in Ni contaminated soil. Moreover, the same treatment modulated the Ni-stress in plants through improvement in antioxidant enzymes (AEs) activities along with improved Ni concentration in the soil and different plant tissues. Correlation and principal component analysis (PCA) further revealed that combined application of metal-tolerant bacterium *Caulobacter* sp. MN13 and zeolite is the most influential strategy in alleviating Ni-induced stress and subsequent improvement in growth, yield, and physio-biochemical attributes of sesame plant.

## 1. Introduction

Global environmental degradation due to anthropogenic activities, particularly, extensive use of heavy metal-bearing chemicals in agriculture, transportation, chemical industry, et cetera, has increased dramatically [1]. Contamination of soil with heavy metals, particularly nickel (Ni), has severely affected plant production in the modern industrial world, which needs to be resolved [2]. Among micronutrients, Ni is essential for optimum plant growth and plays a vital role in nitrogen metabolism, plant senescence, iron (Fe) uptake, and disease resistance [3]. Its required concentration in most plant species is very low (0.05–10 mg/kg^−1^ dry weight), above this concentration, it causes toxicity [4]. As one of the most widely used heavy metals in the world, it is released into the environment mainly through anthropogenic activities such as industrial electroplating, mining and smelting of metals, unwise disposal of Ni-Cd batteries, burning of fossil fuels, paint manufacturing, and Ni-steel and iron alloys [5,6,7,8].

On the other hand, higher concentrations of Ni in soil severely retards important plant processes such as metabolic machinery of plants [9], thereby causing Fe deficiency which ultimately leads to chlorosis and necrosis in plants [10,11]. Elevated levels of Ni in the plant body also cause osmotic imbalance, retarding of photosynthesis, and disruption in cell structure [8]. Besides its high level in the soil, it also causes oxidative damage and initiates overproduction of reactive oxygen species (ROS) in the leaves which further impairs normal cellular functions [12,13,14]. Along with this, an excessive amount of Ni in soil not only deteriorates plant health and productivity but also badly affects its growth and development [8].

Various physicochemical and biological approaches have been employed in successful removal of Ni from the environment; however, eco-friendly biological approaches like bioremediation, phytoremediation, or combination of both, are gaining the attention of the scientific community [15]. These approaches are economical and environment friendly compared to conventional physicochemical technologies [16]. Co-utilization of metal-tolerant bacteria and plant species is a relatively less explored area within this scientific context [17,18]. These bacteria when applied, aggressively colonize the plant roots and promote its growth through an array of mechanisms such as reduced availability and uptake of metals in the rhizosphere via the production of exopolysaccharides (EPS), 1-aminocyclopropane−1-carboxylic acid (ACC) deaminase, siderophores, and metal phosphates [19,20]. The resulting decrease in metal concentrations, in turn, favors better root proliferation and plant growth [17]. In this regard, endophytic bacteria have been proven successful candidates for the bioremediation process, as these microbes can colonize internal plant tissues without being affected by harsh environmental conditions [21,22,23]. Due to the limitations of conventional methods, application of organic and inorganic amendments as an alternative strategy, has been gaining popularity around the globe recently [24,25]. In situ immobilization of soil contaminants, particularly heavy metals, has become one of the most effective technique for minimizing the availability and mobility of hazardous metals [26,27,28,29,30,31,32]. Interestingly, use of zeolite (a naturally occurring microporous aluminosilicate mineral), having plenty of negative charges to accommodate multiple cations for in situ immobilization of metals, has been proven to reduce Ni uptake by plants [33,34]. High surface area, cation exchange capacity (CEC), and porous structure of zeolite make it a suitable candidate for Ni immobilization in the soil [8,35]. In dehydrated form, zeolite becomes porous with pore diameters between 0.3 and 1.0 nm and acts like a molecular sieve that can effectively immobilize pollutants through adsorption [36].

To the best of our knowledge, no report is yet available on the use of sesame plant along with zeolite and endophytic bacteria for the phytoremediation of Ni-contaminated soils and subsequent retrieval effects on the growth, physiology, and biochemistry of sesame. There is also a need to corroborate the beneficial effects of combined application of zeolite and metal-tolerant bacteria to alleviate Ni stress and enhance the growth and grain quality parameters of crops. Therefore, the specific objectives of this study were to: (i) observe the beneficial role of endophytic bacterium (*Caulobacter* sp. MN13) and zeolite (alone and in combination) for Ni immobilization in artificially Ni spiked soil, (ii) test the efficacy of applied amendments on retrieved effects of Ni immobilization as well as improvement in growth and seed quality parameters of sesame, and (iii) evaluate the extent of Ni uptake by different parts of sesame plant.

## 2. Materials and Methods

### 2.1. Acquisition of Endophytic Bacterial Strain and Minimum Inhibitory Concentration (MIC)

Endophytic plant growth-promoting endophytic bacterium *Caulobacter* sp. MN13, previously isolated from the surface-disinfected roots of maize plants [37,38], was used to enhance the growth, production, and physiochemical characteristics of sesame plant. Initially, *Caulobacter* sp. MN13 was grown for 2 days at 28 ± 2 °C using tryptic soya agar (TSA), then streaked on TSA plates, prepared freshly and augmented at 0, 40, 80, 120, 160, and 200 µg mL^−1^ concentrations of Ni (NiSO_4_), until bacterium growth was fully retarded by Ni. Minimal inhibitory concentration (MIC) is that concentration of Ni at which *Caulobacter* sp. MN13 growth got retarded and was measured. In the same way, for inoculation of *Caulobacter* sp. MN13, tryptic soya broth (TSB) tubes (10 mL) amended with different levels of Ni (as described above) were used and kept in a shaker incubator for 5 d (180 rpm, 28 ± 2 °C). Measurement of bacterium growth was taken at 600 nm absorbance on spectrophotometer after 5 d of inoculation.

### 2.2. Analysis of Plant Growth-Promoting (PGP) Attributes of Caulobacter sp. MN13

The endophytic bacterial strain MN13 was characterized regarding various plant growth-promoting activities under normal and Ni (90 µg mL^−1^) stressed conditions. Analysis of PGP traits of selected strain was carried out in triplicates. Auxin was measured colorimetrically both in the absence and presence of tryptophan (0.5%) as precursor. It is expressed as IAA (indole acetic acid) equivalent according to Sarwar et al. [39], method. For determination of ACC-deaminase activity, amounts of ammonia and α-ketobutyrate were measured by following the method of Penrose and Glick [40]. For qualitative measurement of phosphate solubilization, NBRI (National Botanical Research Institute) growth medium was amended with different sources of phosphorus like Ca_3_(PO4)_2_, KH_2_PO_4_, FePO_4_, and AlPO_4_ under normal and Ni-stressed conditions [41]. Siderophore production of strain MN13 was assayed qualitatively using chrome azurol S dyes following Schwyn and Neilands’ [42] method. Qualitative determination of exopolysaccharide and ammonia production was carried out by following the modified methods of Ashraf et al. [43] and Cappuccino and Sherman [44], respectively. Screening of bacterial strains was conducted by inoculating King’s B agar plates amended with glycine (4.4 g L^−1^) to produce hydrogen cyanide (HCN) [45]. For the measurement of oxidase and catalase activities, tetramethyl-p-phenylene diamine (1%) and hydrogen peroxide (H_2_O_2_) (3%) solutions were used, respectively. For analysis of biofilm formation, overnight grown bacterial culture stained with crystal violet (CV) (1%) for 60 min was used, in 96 well microtiter plates. To quantify, the absorbance of destained CV (150 μL) was measured at wavelength of 595 nm [46]. The aggregation stability of the isolate was determined in three replicates according to Madi and Henis [47] under normal and Ni-stressed conditions.

### 2.3. Experimental Design and Treatment Plan

For investigation of interactive effects of bacterial inoculation and zeolite on sesame growth, physiology, yield, and biochemical parameters under Ni-contaminated soil, the present research was conducted in plastic pots in the wire house, at the Institute of Soil and Environmental Sciences (ISES), University of Agriculture, Faisalabad (UAF), Pakistan. The present study was comprised of following four treatments at two levels of Ni, viz., 0 mg Ni kg^−1^ (normal soil) and 90 mg Ni kg^−1^ (Ni-stressed soil) as, (1) control; (2) zeolite; (3) *Caulobacter* sp. MN13; and (4) zeolite + *Caulobacter* sp. MN13.

Nickel sulfate hexahydrate (NiSO_4_·6H_2_O) salt was used to artificially contaminate the soil at the rate of 90 mg Ni kg^−1^ soil and incubated for 30 days before seed sowing. In this pot experiment, 8 kg sieved soil was put into each pot and zeolite was added at the rate of 10 g kg^−1^ soil (*w*/*w*). From the field area, bulk surface soil sample (0–20 cm depth) was collected, dried, grinded, and passed through a sieve of 2 mm for the determination of physicochemical attributes. The texture was sandy clay loam, (typic haplocambid) having pH 7.5, organic matter 7.7 g kg^-1^, and electrical conductivity (ECe) 1.5 dS m^−1^. While soil total N, extractable K, and available P were 0.036%, 158 mg kg^−1^, and 8.8 mg kg^−1^, respectively. The recommended rate of nitrogen, phosphorus and potash (60–40−20 kg) per ha was thoroughly mixed and sesame seeds were sown (5 seeds per pot). The pots were arranged in completely randomized design (CRD) and each treatment was repeated four times. Later on, after germination, each pot was maintained with three plants and irrigated with tap water when required. Measures regarding plant protection and agronomic practices were also carried out.

### 2.4. Preparation of Inoculum

The inoculum of selected bacterium *Caulobacter* sp. MN13 was prepared by transferring a loopful into LB (Luria–Bertani) broth (200 mL) in an Erlenmeyer flask of 500 mL capacity, and *Caulobacter* sp. MN13 was incubated (28 ± 2 °C) in an orbital shaking incubator at 180 rpm for 48 h. For the adjustment of optical density (OD_0.5_) of broth to 535 nm wavelength, a spectrophotometer was used to get a uniform bacterial population (10^9^ CFU mL^−1^), at the time of inoculation.

### 2.5. Treatment of Seeds Before Sowing

Before sowing, surface sterilized sesame seeds (first dipping in 70% ethanol—45 s, then in 5% sodium hypochlorite solution—5 min, and finally washed thrice with distilled water to remove sterilizing agent, if any) were inoculated with broth, after mixing with clay, peat, and sugar solution (10%) [38]. The ratio of slurry comprised of peat and clay was adjusted to 1:1 *w*/*w* [48] After this, seeds were shaken well till a fine coating of slurry containing inoculum appeared on them [17]. Control was treated with sterilized broth containing peat, clay, and sugar solution. Finally, both inoculated and un-inoculated seeds of sesame were dried in shade and sown in the polyethylene-lined pots.

### 2.6. Measurement of Gas Exchange Attributes

Gas exchange parameters such as transpiration rate (E), photosynthetic rate, vapor pressure deficit (VPD), stomatal conductance (gs), and sub-stomatal conductance (Ci) were measured after 45 days of sowing, using fully expanded leaves with portable CIRAS-3 (PP System: Amesbury, MN, USA). These parameters were measured between 9:00 and 11:00 am and values were averaged. For measurement of leaf chlorophyll contents, in other words, SPAD (soil plant analysis development) value, a chlorophyll meter was used (SPAD-502 Minolta: Osaka, Japan) and three consecutive readings (from the leaf tip to leaf blade) were recorded according to the method given by Wellburn [49].

### 2.7. Plant Biomass Measurement

After harvesting, sesame plants were cut into shoots and roots with sterilized sharp knives. Later, segregated plant parts were washed to remove any adhering dust and metal particles using de-ionized water. Afterward, growth and yield attributes of sesame including dry weight of root and shoot, plant height, 1000-grains weight, and number of pods per plant were measured. Dry weight of sesame root and shoot was recorded in triplicate after drying whole plants for 72 h at 65 °C in the oven (Memmert oven 100-800, Memmert GmbH, Schwabach, Germany).

### 2.8. Measurement of Physiological Attributes

Flag leaves (one cm^2^ leaf disc without midrib) were used to determine relative water contents (RWC) along with relative membrane permeability (RMP). The RWC, which is a measure of hydration state of the leaf and its increased value is a symbol of healthy plants, was determined according to Teulat et al.’s [50] method:

(1) $$\mathrm{RWC}\;\left(\%\right)=\frac{\mathrm{Fresh}\;\mathrm{weight}-\mathrm{Dry}\;\mathrm{weight}}{\mathrm{Turgid}\;\mathrm{weight}-\mathrm{Dry}\;\mathrm{weight}}\times 100$$

For RMP measurement, equal pieces of sesame leaves were transferred into test tubes containing deionized distilled water (20 mL) and vortexed for 10 s, and initial electrical conductivity (EC_0_) was recorded. For EC_1_ measurement, these tubes were kept at 4 °C for 24 h, and for EC_2,_ samples were autoclaved at 121 °C for 20 min_._ Calculation of percent RMP was done using formula given below by Yang et al. [51]:

(2) $$\mathrm{RMP}\;\left(\%\right)=\frac{EC_{1}-EC_{0}}{EC_{2}-EC_{0}}\times 100$$

### 2.9. Grain Biochemical and Micronutrients Analysis of Shoot

Biochemical parameters (ash, protein, fats, and fiber) of sesame seeds were determined according to the standard protocols. For ash measurement, one-gram grain sample was taken in the crucible and placed in a muffle furnace at 550 °C until the appearances of gray-white ash [52]. The percent ash was measured by using formula given below:

(3) $$\mathrm{Ash}\;\left(\%\right)=\frac{\mathrm{Weight}\;\mathrm{of}\;\mathrm{ash}\;}{\mathrm{Weight}\;\mathrm{of}\;\mathrm{sample}\;}\times 100$$

Protein contents of grain samples were spectrophotometrically determined according to the Bradford colorimetric method [53], while fat contents were spectrophotometrically measured at 660 nm absorbance [54] and calculated by using the following formula:

(4) $$\mathrm{Fat}\;\left(\%\right)\;=\frac{\mathrm{Weight}\;\mathrm{of}\;\mathrm{ether}\;\mathrm{extract}\;}{\mathrm{Weight}\;\mathrm{of}\;\mathrm{sample}\;}\times 100$$

The fiber contents were measured following the methods of The Association of Official Analytical Chemists (AOAC) [52]. Determination of Fe, Zn, Cu, and Mn in the sesame shoot was carried out by digesting a known weight of shoot samples in a di-acid mixture having the ratio 2:1 (HNO_3_:HClO_4_) [55]. The filtrates were run on an atomic absorption spectrophotometer to measure the concentration.

### 2.10. Measurement of Antioxidant Enzymes (AEs)

The fresh-frozen leaves of sesame plants were homogenized in an ice-cold solution (pH 7) of potassium phosphate buffer (200 mM) and ethylenediaminetetraacetic acid (EDTA) (100 mM). Cakmak and Marschner [56] method was followed to measure catalase (CAT) activity spectrophotometrically by measuring reduction in absorbance at 240 nm, due to the H_2_O_2_ elimination from reaction mixture. Ascorbate peroxidase (APX) activity was measured by recording reduction in absorbance at 290 nm, due to oxidation of ascorbate [57]. Methods of Foyer and Halliwell [58], and Aebi and Bergmeyer [59], were followed for the measurement of glutathione reductase (GR) and glutathione peroxidase (GPX), respectively. While for the determination of glutathione-S-transferase (GST) and superoxide dismutase (SOD) activities spectrophotometrically, according to Habig et al. [60] and Roth and Gilbert [61] methods, respectively, the reaction mixture was monitored at λ 340 nm and 420 nm, respectively.

### 2.11. Persistence of Caulobacter sp. MN13 in the Rhizospheric Soil and Parts of Sesame Plant

The persistence of endophytic inoculant strain in the rhizospheric soil, and tissues (root and shoot) of sesame plant was determined by dilution plate counting method. Saline buffer 0.85% (*w*/*v*) was used for suspension preparation. Samples of rhizosphere soil, and root and shoot tissues of sesame (2 g) were obtained and homogenized by mixing with saline buffer (10 mL) and agitating (160 rpm) for 20 min at room temperature. After settling suspensions, serial dilutions up to 10^−4^ were plated onto TSA plates and incubated at 28 ± 1 °C for 48 h. To determine the colony forming unit (CFU) per gram of dry soil/tissue mass, the colonies were counted. Twenty-five colonies were randomly selected from each treatment and for their identification as inoculant strain, restriction fragment length polymorphism (RFLP) analysis of the 16S–23S rRNA intergenic spacer (IGS) region was conducted [38].

### 2.12. Chemical Analysis of Metal

Finally, dried plant parts were crushed and sieved through 0.5 mm sieve for Ni determination. Total Ni concentration in soil, root, shoot, and grain samples was measured using AAS (atomic absorption spectrophotometer—Aanalyst 100, PerkinElmer Inc.: Waltham, MA, USA) by following Lindsay and Norvell’s [62] method. Bioavailable Ni concentration (diethylenetriaminepentaacetic acid (DTPA) Ni) in soil was determined along with its root to shoot translocation.

### 2.13. Statistical Analysis of Data

Data regarding physiological, and biochemical parameters as well as growth and yield of sesame plant, were subjected to statistical analysis through analysis of variance (ANOVA) test [63] by using software SPSS (version 22, IBM: Armonk, NY, USA). Principal component analysis (PCA) was performed using R studio software. The treatment means were compared by DMRT (Duncan’s multiple range test) at *p* ≤ 0.05. Relationships among different attributes of sesame plant was analyzed using Spearman’s correlation coefficient at 95% confidence interval.

## 3. Results

### 3.1. MIC and PGP Characteristics

The MIC of Ni for *Caulobacter* sp. MN13 was tested on TSA medium. It was found that the tested strain was able to grow at 160 µg Ni mL^−1^ (Supplementary Table S1), and possessed various PGP activities such as ACC-deaminase, IAA production, phosphate solubilizing activities, siderophore, exopolysaccharides, HCN, NH_3_ production, catalase, oxidase, aggregation, and biofilm formation (Table 1).

### 3.2. Agronomic Parameters

Combined application of endophytic bacterial strain (*Caulobacter* sp. MN13) and inorganic amendment (zeolite) improved all agronomic parameters including plant height, root and shoot dry weight, number of pods plant^−1^, and 1000-grain weight in comparison to the control treatment (Table 2). There was an increase in all the agronomic parameters recorded with the sole application of *Caulobacter* sp. MN13 or zeolite. However, the maximum increase was observed with the combined application of *Caulobacter* sp. MN13 and zeolite. For example, an increase of 28 and 51% in plant height was observed with the combined application of *Caulobacter* sp. MN13 and zeolite under normal and Ni stressed soil, respectively, as compared to the control (Table 2). The same treatment (*Caulobacter* sp. MN13 and zeolite) increased root dry weight by 35.5 and 44.7%, shoot dry weight by 22 and 41%, respectively, under normal and Ni contaminated soil, as compared to their respective control treatments. Under normal and Ni stressed soil, the maximum increase of 41 and 65% in 1000-grain weight and 38 and 54% in the number of pods plant^−1^ in comparison to the control was observed with the combined application of *Caulobacter* sp. MN13 and zeolite.

### 3.3. Gaseous Exchange Attributes

Gaseous exchange attributes of sesame plant showed a substantial rise with the applied endophytic bacterial strain and zeolite as compared to the control (Table 3). The maximum and a significant increase in the photosynthetic rate (CO_2_ assimilation rate) of sesame plant was observed with the combined application of *Caulobacter* sp. MN13 and zeolite and these were increased up to 14 and 20% under normal (0 mg Ni kg^−1^) and Ni stressed soil (90 mg Ni kg^−1^) as compared to the control. It was followed by the sole application of *Caulobacter* sp. MN13 which enhanced photosynthetic rate by 9 and 16%, respectively, under normal and Ni contaminated soil. Data regarding the transpiration rate (E) showed a similar trend with the application of *Caulobacter* sp. MN13 along with zeolite under normal as well as Ni stressed soil in comparison to the control. Endophytic bacteria applied with zeolite in Ni-contaminated soil increased transpiration rate by 49%, as compared to the control, however, under normal soil conditions increment was up to 37%. A similar trend was observed in stomatal (gs) and sub-stomatal conductance (Ci). Among all the treatments, the combined application of *Caulobacter* sp. MN13 and zeolite increased stomatal conductance by 27 and 33% under normal and Ni stressed soil, respectively, as compared to the control. The same treatment caused a significant increase of 19 and 32% in sub-stomatal conductance, as compared to the control under normal and Ni contaminated soil, respectively. In the case of VPD, combined application of *Caulobacter* sp. MN13 and zeolite caused an increase of 29 and 41% in comparison to the control under normal and Ni spiked soil, respectively. In the same way, the combined application of endophytic bacterial strain and zeolite caused the maximum but non-significant increase (24 and 40%) in chlorophyll contents under both normal and Ni stressed soil as compared to the control (Table 3).

### 3.4. Physiological Measurements

**Figure 1:**
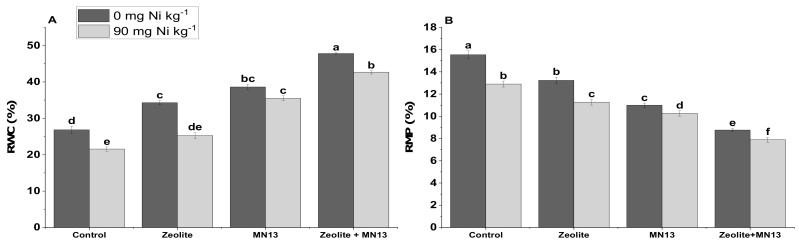
(**A**) Relative water content (RWC) and (**B**) relative membrane permeability (RMP) of sesame plants by different treatments applied under normal and Ni stressed soil conditions. Values sharing the same letter(s) in bars are statistically non-significant with each other at *p* <0.05; the values are mean ± standard error (*n* = 4).

Relative water contents were significantly increased while RMP decreased with both individual and combined application of *Caulobacter* sp. MN13 and zeolite in both normal and Ni stressed soil (Figure 1). However, the maximum increase was only observed with the combined application of *Caulobacter* sp. MN13 and zeolite. The maximum increase (78 and 87%) in RWC under normal as well as Ni stressed soil was observed with the combined application of *Caulobacter* sp. MN13 and zeolite as compared to the control (Figure 1). It was followed by the sole application of *Caulobacter* sp. MN13 and zeolite which increased RWC by 43 and 27% under normal soil conditions and by 64 and 17% under Ni stressed soil, respectively, in comparison with the control. A similar but decreasing trend was observed in the case of RMP. The same treatment showed a decrease of 44% in RMP under normal soil and 38% under Ni stressed soil, as compared to the control.

### 3.5. Activities of AEs

The concentration of various AEs such as glutathione reductase (GR), reduced glutathione (RG), glutathione peroxidase (GPX), ascorbate peroxidase (APX), glutathione S transferase (GST), catalase (CAT), and superoxide dismutase (SOD) was significantly enhanced in Ni-stressed soils (90 mg Ni kg^−1^), as compared to the normal soils (0 mg Ni kg^−1^) (Table 4). However, the activities of all these enzymes (RG, GPX, GR, GST, APX, CAT, and SOD) were significantly reduced with the application of zeolite and *Caulobacter* sp. MN13, alone and in combination, as compared to the control treatment (Table 4).

### 3.6. Grain Biochemical and Micronutrients Analysis of Shoot

Biochemical attributes of grain (fats, protein, fiber, and ash contents) were improved under all the treatments compared to their respective control under normal as well as in Ni stressed conditions (Table 5). However, combined application of zeolite and *Caulobacter* sp. MN13 caused the maximum increase in ash, protein, fiber, and fat contents of sesame grains, under both normal and Ni contaminated soils, as evident from (Table 5). Under Ni stressed soil, the combined application of *Caulobacter* sp. MN13 and zeolite caused an increase of 41, 19, 24, and 23% in ash, protein, fiber, and fat contents in the grains of sesame, respectively, as compared to the control.

**Figure 2:**
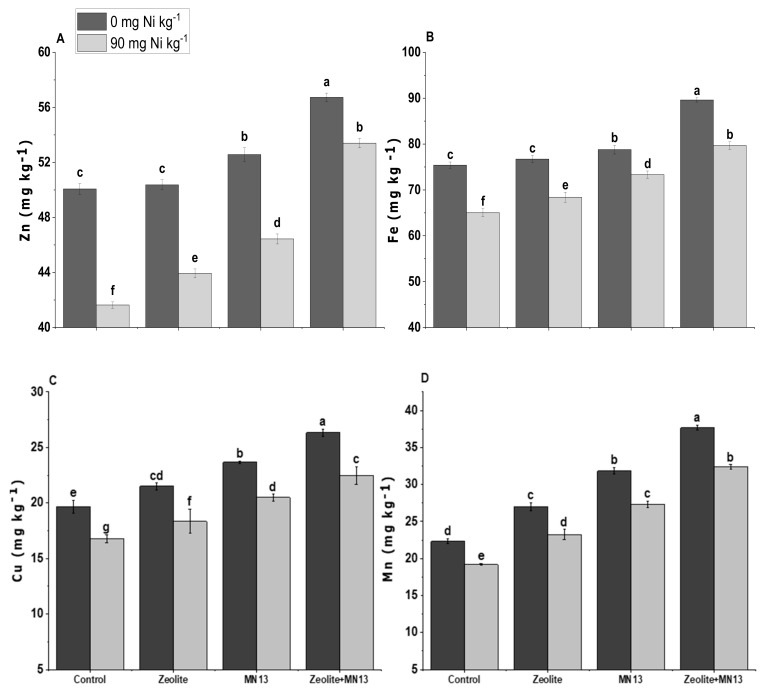
Micronutrients Zn (**A**), Fe (**B**), Cu (**C**), and Mn (**D**) content of sesame shoots by different treatments applied under normal and Ni stressed soil conditions. Values sharing the same letter(s) in bars are statistically non-significant with each other at *p* < 0.05; the values are mean ± standard error (*n* = 4).

In the present study, Zn and Fe concentrations in sesame shoot were significantly enhanced with the application of *Caulobacter* sp. MN13 and zeolite applied alone or in combination as compared to the control under normal as well as Ni stressed soil (Figure 2). Combined application of *Caulobacter* sp. MN13 and zeolite, however, caused the maximum increase in both Zn and Fe concentration as compared to all other treatments. The combined application of *Caulobacter* sp. MN13 and zeolite caused the maximum increase in Fe concentration under normal (18%) as well as Ni stressed soil (19%) as compared to the control. The same treatment caused an increase of 13 and 28% in Zn concentration under normal and Ni contaminated soil over the control (Figure 2). In the case of Cu and Mn, the combined application of *Caulobacter* sp. MN13 and zeolite caused an increase of 34 and 69% increase in Cu and Mn contents of sesame shoot as compared to the control treatment under Ni contaminated soil (90 mg kg^−1^).

### 3.7. Persistence of Inoculant Strain in Rhizosphere Soil, and Sesame Root and Shoot Tissues

**Figure 3:**
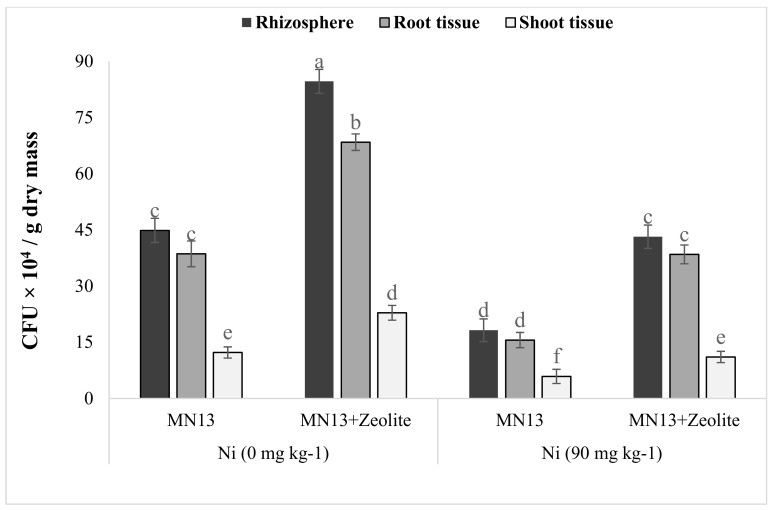
Persistence of the endophytic bacterial strain MN13 in the rhizosphere and presence in root and shoot extracts of sesame grown in Ni stressed soil conditions. Bars sharing the same letters do not differ significantly with each other at *p* < 0.05. The values are mean ± S.D. (*n* = 4). MN13: *Caulobacter* sp.

The inoculant strain MN13 effectively colonized the rhizosphere and was endophytic in the parts of sesame plant vegetated in normal and Ni contaminated soils (Figure 3). Zeolite application enhanced bacterial colonization of the rhizosphere, particularly in the Ni contaminated environment. Similarly, endophytic population assessed from root and shoot extracts was enhanced in the presence of zeolite under both normal and in Ni stress. Maximum CFU g^−1^ dry weight recovered from the rhizosphere (8.46 × 10^5^), root interior (6.83 × 10^5^), and shoot interior (2.28 × 10^4^) was the combined application of zeolite and MN13.

### 3.8. Ni Concentration in Soil, Root, Shoot, and Grain of Sesame

**Figure 4:**
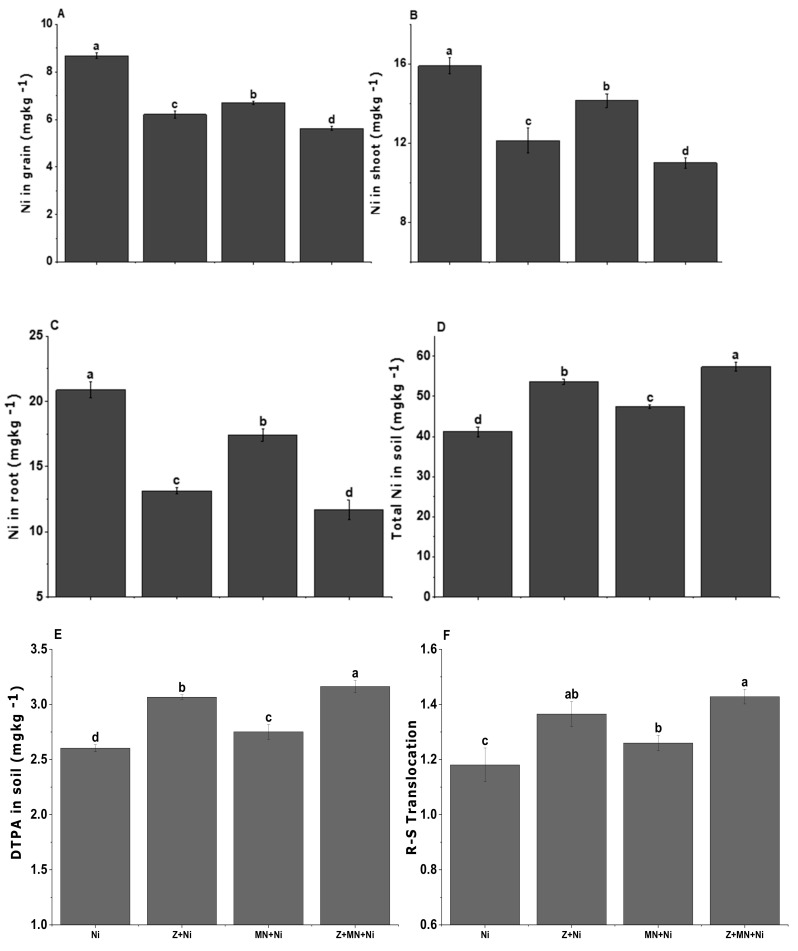
Ni concentration in different parts grain (**A**), shoot (**B**), root (**C**) of sesame plants and in soil (**D**,**E**) and its R-S translocation (**F**) with different treatments applied under normal and Ni stressed soil conditions. Values sharing the same letter(s) in bars are statistically non-significant with each other at *p* < 0.05; the values are mean ± standard error (*n* = 4).

Data regarding Ni concentration in shoot and grain samples of sesame showed a significant reduction while showing an increase in soil and root samples due to the combined application of *Caulobacter* sp. MN13 and zeolite (Figure 4). The maximum Ni uptake was observed under control conditions where neither bacteria nor zeolite was applied. The minimum uptake in shoot and grains was observed by cumulative application of *Caulobacter* sp. MN13 and zeolite and these were 57 and 55.6% less compared to the control. The same treatment showed an increase of 29.5 and 31% in Ni concentration in sesame roots and soil, compared to the treatment set as control where no amendment was added. In the case of Ni root-shoot translocation, the combined application of *Caulobacter* sp. MN13 and zeolite revealed the minimum translocation.

### 3.9. Correlation among Different Attributes of Sesame Plants and Principal Component Analysis (PCA)

**Figure 5:**
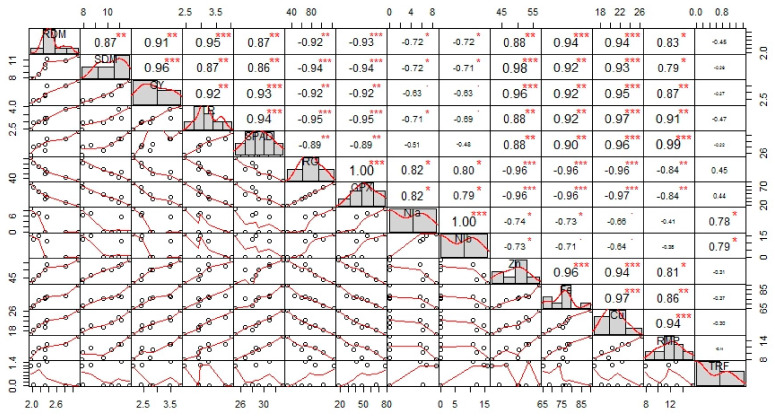
Correlation among different attributes of sesame plant by different treatments as control, (1) Ni 0 mg kg^−1^ (2) Ni 90 mg kg^−1^; zeolite, (3) Ni 0 mg kg^−1^ (4) Ni 90 mg kg^−1^; *Caulobacter* sp. MN13, (5) Ni 0 mg kg^−1^ (6) Ni 90 mg kg^−1^; and *Caulobacter* sp. MN13+ zeolite, (7) Ni 0 mg kg^−1^ (8) Ni 90 mg kg^−1^. The abbreviations are as RDM: root dry biomass, SDM: shoot dry biomass, GY: grain yield, CO_2_ AS: CO_2_ assimilation rate, TR: transpiration rate, SPAD: chlorophyll contents, RG: reduced glutathione, GPX: glutathione peroxidase, Nia: Ni in grain, Nib: Ni in shoot, Zn: Zn in shoot, Fe: Fe in shoot, Cu: Cu in shoot, Mn: Mn in shoot, and TRF: translocation factor. *, 0.7–0.8; **, 0.8–0.9; ***, 0.9–1.0.

**Figure 6:**
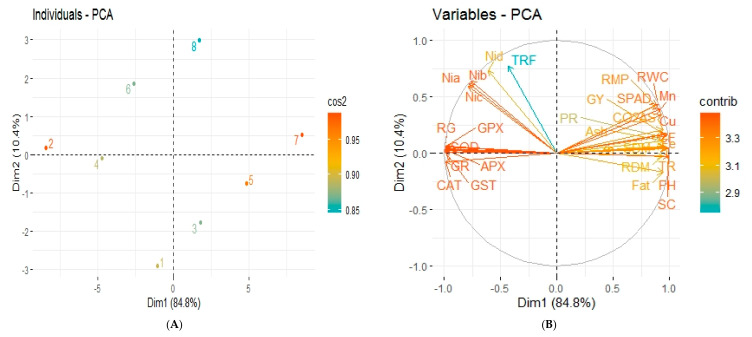
Principal component analysis (PCA) showing score plots (**A**) and loading plots (**B**) of different attributes of sesame plants. Score plots (A) represents separation of treatments as control, (1) Ni 0 mg kg^−1^ (2) Ni 90 mg kg^−1^; zeolite, (3) Ni 0 mg kg^−1^ (4) Ni 90 mg kg^−1^; *Caulobacter* sp. MN13, (5) Ni 0 mgkg^−1^ (6) Ni 90 mg kg^−1^; *Caulobacter* sp. MN13 + zeolite, (7) Ni 0 mg kg^−1^ (8) Ni 90 mg kg^−1^.The abbreviations are as PH: plant height, RDM: root dry biomass, SDM: shoot dry biomass, GY: grain yield, CO_2_AS: CO_2_ assimilation rate, TR: transpiration rate, SC: stomatal conductance, SPAD: chlorophyll contents, RG: Reduced glutathione, GPX: Glutathione peroxidase, GR: Glutathione reductase, GST: Glutathione S transferase, APX: Ascorbate peroxidase, CAT: catalase, SOD: superoxide dismutase, Ash: Ash %, CF: crude fiber %, PR: protein %, Fat: fat%, Nia: Ni in grain, Nib: Ni in shoot, Nic: Ni in root, Nid: Ni in soil, Zn: Zn in shoot, Fe: Fe in shoot, Cu: Cu in shoot, Mn: Mn in shoot, RWC: relative water contents, RMP: relative membrane permeability, and TRF: translocation factor.

Highly significant (*p*-value < 0.01) and mostly positive correlation was observed among different attributes of sesame plants by separate and combined application of *Caulobacter* sp. MN13 and zeolite under normal and Ni stressed soil conditions (Figure 5). Principal component analysis (PCA) of all the treatments was performed on the basis of studied attributes of sesame plant as presented by score and loading plots (Figure 6). Remarkable results were obtained from score plots of PCA, showing great variation among different treatments applied on sesame plants under normal and Ni stressed soil conditions, with the first two principal components explaining 95.2% of variability (Figure 6). Maximum coordinates on the score plot of PCA was obtained by the combined application of *Caulobacter* sp. MN13 and zeolite under normal soil conditions (Ni 0 mg kg^−1^), revealing it to be the most effective and efficient treatment in improving the overall growth, physiochemical characteristics, and yield of sesame plants, followed by the sole application of *Caulobacter* sp. MN13 under normal soil conditions (Figure 6).

## 4. Discussion

Nickel contamination of soil is an important economical and serious environmental and health issue in developing countries of the world including Pakistan. Although numerous methods of soil remediation have been employed, the extent to which soil and water environments are polluted, has become highly hazardous for humans. Therefore, it is the dire need of the time to implement site-specific remediation methods that are economical and ecofriendly. In this regard, the present study was designed and carried out to evaluate the effect of Ni contaminated soil on the growth, health, yield, physiological, and biochemical attributes of sesame plants and its remediation through the application of either endophytic bacteria (*Caulobacter* sp. MN13), zeolite, or both.

In this study, various agronomic parameters (plant height, root and shoot dry weight, number of pods plant^−1^, and 1000-grain weight) were significantly improved with the combined application of *Caulobacter* sp. MN13 and zeolite, under normal as well as Ni contaminated soils. However, in the absence of *Caulobacter* sp. MN13 or zeolite, all the agronomic attributes of sesame were severely affected under Ni stressed soil conditions, as evident from the data presented in Table 3. The observed growth reduction might be due to the excess amount of Ni and its subsequent interference with various biochemical and metabolic processes such as synthesis of chlorophyll and protein [3,9]. This premise is supported by a significant reduction in chlorophyll content observed in the present study (Table 3). Significant enhancement of growth and yield parameters, by sole or combined application of endophytic bacteria and zeolite, might be due to the capacity of endophytes to alleviate phytotoxicity in metal contaminated soils through mechanisms such as (i) endophytes metal ions efflux from cell, (ii) helping in the transformation of metals from more toxic forms to less toxic ones, (iii) sequestering metals in intracellular polymers and on cell surfaces, and (iv) causing either precipitation/adsorption/desorption, biomethylation of metals, or both [31,64]. It may also be due to the endophytic assistance in the remediation of metal contaminated soil through nutrient acquisition and improved cell elongation [22,65,66]. Endophytes prevail inside the plant roots, colonize root cells, and enhance the root infrastructure resulting in improved nutrient uptake and plant growth. Furthermore, endophytic bacteria not only minimize the disease severity by suppressing pathogens but also exude a plethora of plant growth regulators (IAA, ACC deaminase, siderophores), which help improve plant growth under biotic and abiotic stress conditions such as heavy metals’ stress as observed in the present study (Table 1). The findings of the present work are in accordance with earlier studies [17,18,38,66,67,68] that application of bacteria has positive effects to alleviate metal stress with subsequent plant growth promotion. Also, a three-dimensional porous structure of zeolite acts as a sieve for metals and reduces their availability for plants by converting their extractable forms to unexchangeable forms and absorption on zeolite [35,69]. Further, a slow and steady release of micro and macronutrients (nitrogen—N, potassium—K, phosphorus—P, and iron—Fe) from zeolite due to applied bacteria, may have helped to improve the root and shoot growth of plants and hence ameliorated the harmful effects of Ni stress [17,70]. The enhancement of agronomic parameters of sesame plant is supported by the fact that significant positive and negative correlations were observed among Ni concentration in soil and plant tissues and the studied agronomic parameters (Figure 5 and Figure 6).

In this study, plant physiological parameters (internal CO_2_ concentration, stomatal conductance, VPD, etc.) indicated that photosynthetic and transpiration rate, and chlorophyll contents were severely affected under Ni stress, compared to the normal soil (Table 4). Reduction in photosynthetic and transpiration rates might be due to the formation of a decreased number of photosynthetic pigments under Ni stressed conditions [18,68]. In addition to this, chlorophyll contents of leaf tissues were significantly reduced under Ni stressed soil, which might be due to the inhibition of chlorophyll biosynthesis [10]. According to Gajewska et al. [71], chlorophyll biosynthesis might be reduced due to the damage caused to the ultra-structure of chlorophyll molecules via the entrapment of Ni in place of Mg^2+^, thereby causing a reduction in the efficiency of enzymes involved. Several other researchers have also reported a decrease in chlorophyll contents due to the deficiency of Fe and Mg in plants exposed to heavy metals stress [72,73]. Along with this, phytotoxicity caused by heavy metals, produces ROS, thereby causing membrane lipid peroxidation in plants [71,74,75,76].

However, in the present study, cumulative application of zeolite and *Caulobacter* sp. MN13 caused the highest increase in physiological parameters, as compared to the normal and Ni stressed soil. This increase might be attributed to the plant-microbe interactions which help improve micro- as well as macronutrients through various mechanisms such as the exudation of siderophores [77,78,79,80]. This premise is supported by the data presented in (Figure 2), showing a significant increase in the concentration of Fe and Zn in sesame tissues. The improved availability of micronutrients (Fe and Zn) might have increased the activity of enzymes involved in various physiological functions [81], thereby improving the recorded physiological parameters of sesame. The improvement in physiological parameters may also be due to the binding of toxic metal ions with zeolite which decreased the availability of Ni to the crop plants [82,83,84], and ultimately improved physiological function by reducing the toxic effect of Ni. Can et al. [82] used zeolite for the removal of Ni, copper (Cu), and cobalt (Co) in batch and column experiments and found a significant reduction in the availability of heavy metals. This was further supported by correlation and PCA (Figure 5 and Figure 6), that suggested the combined application of bacteria and zeolite was the most influential treatment in improving growth, yield, and physiological parameters of sesame.

In the present study, RWC was significantly decreased under Ni stressed soil, without the application of any amendment. As mentioned earlier, high amounts of Ni had a toxic effect on various biochemical functions of crop plants [75]; while *Caulobacter* sp. MN13 and zeolite application, alone or in combination, reduced this stress. The maximum RWC was observed when *Caulobacter* sp. MN13 and zeolite were used collectively. These findings are supported by earlier studies [18,20,37]. Under Ni stress, RMP was increased which is in line with other researchers who reported a significant increase in RMP under abiotic stresses in different crop plants [85]. In the present study, an increase in RWC and decrease in RMP with the application of *Caulobacter* sp. MN13 and zeolite might be due to the positive role of endophytic bacteria in improving plant growth and yield parameters, and that of zeolite in decreasing Ni stress by sorption and complexation mechanisms [66,67,86].

Under abiotic stress, increased production of ROS in plants poses oxidative stress, resulting in inactivation of enzymes, nucleic acid damage, oxidation of protein, programmed cell death activation, and peroxidation of lipids [76,87]. To cope with oxidative stress, the plants actively secrete various types of AEs that regulate the stress condition to normal for optimum plant growth [88]. In the present study, the production of antioxidants (RG, GPX, GR, GST, APX, CAT, and SOD) was enhanced under Ni stress which might be a metabolic response against oxidative stress caused. Cumulative application of zeolite and *Caulobacter* sp. MN13 significantly decreased the production of AEs which might be due to the role of zeolite in the immobilization of Ni in soil (Table 5). This premise is supported by elevated levels of Ni in soil as revealed by the post-harvest soil analysis in the present study (Figure 4). Moreover, the endophytic bacterial strain used in the present study can produce exopolysaccharides that might have immobilized Ni in soil and ultimately reduced Ni availability to the crop plants [23,37]. Earlier, Castaldi et al. [89] found that zeolite can bind the metal and is alkaline in nature, which increases soil pH, thereby reducing metal uptake. Earlier, it has been found that endophytes are capable of altering availability and toxicity of heavy metals to plants through siderophores production, and mobilization of metal phosphates and organic acids [20,90,91].

Application of *Caulobacter* sp. MN13 along with zeolite also improved the plant biochemical parameters (ash, fiber, protein, etc.). Zeolite is well-known as a soil conditioner and improves the physicochemical properties of soil and neutralizes metals through exchange of cations in its 3-D cage-like structural sites [36,89]. It has also been found that bacteria are the most important organisms having great potential for detoxification and immobilization of metal pollutants [31,67]. Microbes exude various kinds of functional groups and hormones, having a negative charge and potential to bind positively charged metals, thereby reducing their bioavailability and ultimately toxicity of the metal. Furthermore, microbes help in improving nutrient availability in the rhizosphere which competes with metal pollutants, thereby reducing metal uptake into the crop plants [92].

This study revealed high proportion of Ni accumulation in plants of the control (no amendment) treatment, which might be due to its high availability in contaminated soil. Earlier, it has been found that plant uptake of Ni depends upon its concentration, acidity and composition of soil organic matter, plant breakdown process, presence of other metals, soil texture, and composition and structure of soil minerals [93].

The bacterium (*Caulobacter* sp. MN13) used in the study was stress tolerable, having the ability to secrete exopolysaccharides that can bind heavy metals, thereby reducing their availability in the soil and ultimately reduced plant uptake. Moreover, the higher accumulation of micronutrients in the shoot portion may also be the reason for lower accumulation of Ni in the shoot and subsequent translocation to the grain portion of the plant. The results regarding reduced uptake of metal in the present study are in good agreement with [17,37]. Various researchers have also found that endophytes are capable of altering toxicity and availability of heavy metals to the plants through the production of siderophores and mobilization of organic acids and metal phosphates [18,87,90,92,94,95]. Moreover, microbial inoculation helps to enhance plants’ ability to survive under stressful conditions [96]. The application of *Caulobacter* sp. MN13 in combination with zeolite as a win-win strategy in the present study might explain the reason for better performance of plants grown under Ni stressed soil.

## 5. Conclusions

An increasing amount of toxic heavy metals, particularly Ni, in the soil environment has adversely affected plant growth, biomass, and yield. Multiple remediation technologies are being employed to alleviate metal phytotoxicity and restore a healthy functioning soil. In this study, combined application of chemical and biological amendments proved significantly effective in improving soil environment and plant growth by immobilizing metals in metal-contaminated soil. Application of zeolite enhanced plant biochemical, agronomic, and physiological attributes, and inoculation of *Caulobacter* sp. MN13 further enhanced overall health, growth, biomass, and yield of sesame plant under Ni-stressed conditions. In the present study, application of zeolite along with endophytic bacteria revealed synergistic effects on the phytoimmobilization efficiency of sesame plant under Ni contaminated soil conditions. This avenue of research will help to remediate metal contaminated soils resulting in improved plant growth and health in an economical and environment friendly green way across a broad range of climatic conditions. In future, the combined effect of endophytic bacteria and zeolite needs be tested under field conditions involving different plant species and variable climate conditions in order to warrant their practical application.

## Figures and Tables

**Table 1:** In Vitro plant growth promoting (PGP) characteristics of *Caulobacter* sp. MN13 under normal and Ni-stressed conditions.

PGP Traits	Ni (0 µg mL^−1^)	Ni (90 µg mL^−1^)
IAA (without L-Tryp)	7.85 ± 0.49	2.64 ± 0.36
IAA (With L-Tryp)	15.25 ± 1.08	9.14 ± 0.98
ACC deaminase activity	+	+
P-solubilization		
Ca_3_(PO4)_2_	−	−
KH_2_PO_4_	+	+
FePO_4_	+	+
AlPO_4_	+	+
Siderophore production	+	+
Exopolysaccharides production	+	+
HCN production	−	−
NH_4_ production	+	+
Catalase	+	+
Oxidase	−	−
Biofilm formation		
OD (600 nm)	0.94 ± 0.04	0.64 ± 0.03
Biofilm (595 nm)	0.29 ± 0.02	0.20 ± 0.02
Aggregate stability	36.90 ± 2.52	25.07 ± 0.82

The values are mean ± standard error (*n* = 4). IAA: indole-3-acetic acid (μg mL^−1^); L-Tryp: L-tryptophan; ACC: 1-aminocyclopropane-1-carboxylic acid; HCN: hydrogen cyanide; OD: optical density, (+): activity is present; (−): activity is absent.

**Table 2:** Effect of *Caulobacter* sp. MN13 and zeolite on growth and yield of sesame grown in normal and Ni contaminated soil.

Treatments	Ni (mg kg^−1^)	Plant Height (cm)	Root Dry Biomass (g plant^−1^)	Shoot Dry Biomass (g plant^−1^)	Number of Pods plant^−1^	1000-Grains Weight (g)
Control	0	43.01 ± 1.91 c	2.31 ± 0.162 bc	9.67 ± 0.09 bc	15.66 ± 1.7 cd	2.75 ± 0.28 ab
	90	32.80 ± 1.42 de	1.97 ± 0.087c	7.88 ± 0.25 d	12.33 ± 1.69 d	2.11 ± 0.19 c
Zeolite	0	48.03 ± 1.68 b	2.32 ± 0.162 bc	10.31 ± 0.12 ab	17.33 ± 0.5 a–d	2.85 ± 0.21 ab
	90	37.56 ± 1.98 d	2.03 ± 0.154 c	7.90 ± 0.5 d	16.0 ± 2.5 bc	2.41 ± 0.08 bc
*Caulobacter* sp. MN13	0	52.03 ± 1.88 ab	2.81 ± 0.175 ab	10.87 ± 1.02 ab	18.66 ± 1.9 a–c	3.47 ± 0.15 b
	90	40.54 ± 1.72 cd	2.17 ± 0.159 c	8.57 ± 0.73 cd	16.96 ± 2.3 a-d	2.44 ± 0.05 bc
Zeolite + *Caulobacter* sp. MN13	0	55.03 ± 1.94 a	3.13 ± 0.202 a	11.80 ± 1.18 a	21.66 ± 2.1 a	3.88 ± 0.23 a
	90	49.62 ± 1.70 b	2.85 ± 0.166 bc	11.11 ± 1.23 ab	19.00 ± 2.04 ab	3.49 ± 0.09 b

Values sharing the same letter(s) in a column are statistically non-significant with each other at *p* < 0.05; the values are mean ± standard error (*n* = 4).

**Table 3:** Effect of *Caulobacter* sp. MN13 and zeolite on physiological parameters of sesame in normal and Ni contaminated soil.

Treatments	Ni (mg kg^−1^)	CO_2_ assimilation Rate (µmol CO_2_ m^−2^ s^−1^)	Transpiration Rate (µmol CO_2_ m^−2^ s^−1^)	Stomatal Conductance (µmol CO_2_ m^−2^ s^−1^)	Substomatal Conductance (µmol CO_2_ m^−2^ s^−1^)	Vapor Pressure Deficit (KPa)	Chlorophyll Contents (SPAD value)
Control	0	9.03 ± 0.32 bc	2.93 ± 0.95 c	224.6 ± 5.59 b–d	276.43 ± 4.21 bc	2.36 ± 0.21 b–d	26.8 ± 1.11 cd
	90	8.16 ± 0.22 c	2.45 ± 0.66 e	180.6 ± 4.41 d	240.6 ± 4.78 c	1.81 ± 0.09 e	22.1 ± 0.75 d
Zeolite	0	9.6 ± 0.39 a–c	3.26 ± 0.29 bc	239.0 ± 6.11 a–c	288.33 ± 5.98 ab	2.57 ± 0.61 a–c	29.0 ± 1.27 bc
	90	9.2 ± 0.53 bc	2.93 ± 0.91 c	191.3 ± 4.33 cd	272.3 ± 5.06 bc	1.86 ± 0.15 de	27.9 ± 0.93 b–d
*Caulobacter* sp. MN13	0	9.93 ± 0.33 ab	3.82 ± 0.48 ab	255.6 ± 5.82 ab	299.0 ± 4.73 a	2.94 ± 0.34 ab	30.9 ± 0.69 ab
	90	9.5 ± 0.37 a–c	2.98 ± 0.99 c	204.6 ± 4.99 b–d	276.7 ± 5.65 bc	2.13 ± 0.19 c–e	28.6 ± 1.10 b–d
Zeolite + *Caulobacter* sp. MN13	0	10.3 ± 0.48 a	4.03 ± 1.11 a	285.0 ± 6.08 a	330.3 ± 6.33 a	3.04 ± 0.43 a	33.3 ± 1.22 a
	90	9.8 ± 0.61 a–c	3.65 ± 0.44 bc	240.0 ± 4.37 a–c	318.7 ± 5.87 ab	2.55 ± 0.19 a–c	30.9 ± 0.97 ab

Values sharing the same letter(s) in a column are statistically non-significant with each other at *p* < 0.05; the values are mean ± standard error (*n* = 4). SPAD: Soil Plant Analysis Development.

**Table 4:** Effect of *Caulobacter* sp. MN13 and zeolite on antioxidant assays of sesame plant in normal and Ni contaminated soil.

Treatments	Ni (mg kg^−1^)	RG (nmol g^−1^ FW)	GPX (nmol min^−1^ mg^−1^)	GR (nmol min^−1^ mg^−1^)	GST (µmol min^−1^ mg^−1^)	APX (nmol min^−1^ mg^−1^)	CAT (nmol min^−1^ mg^−1^)	SOD (nmol min^−1^ mg^−1^)
Control	0	76.0 ± 1.16 cd	48.1 ± 0.85 cd	19.8 ± 0.6 cd	94.0 ± 1.27 c	39.0 ± 1.4 cd	13.9 ± 0.313 c	142.7 ± 3.98 cd
	90	125.0 ± 1.19 a	78.5 ± 1.19 a	34.0 ± 0.71 a	146.0 ± 1.34 a	63.7 ± 2.03 a	22.0 ± 0.521 a	221.3 ± 8.31 a
Zeolite	0	60.7 ± 0.48 e	37.5 ± 0.66 ef	15.3 ± 0.41 e	72.0 ± 1.16 d	31.3 ± 1.31 e	10.4 ± 0.181 de	115.7 ± 1.41 e
	90	103.7 ± 0.98 b	66.2 ± 1.14 b	27.5 ± 1.04 b	124.2 ± 1.10 b	56.2 ± 1.27 b	17.7 ± 0.495 b	190.7 ± 2.41 b
*Caulobacter* sp. MN13	0	46.0 ± 0.41 f	28.3 ± 0.57 f	10.7 ± 0.09 f	54.5 ± 0.48 e	22.3 ± 0.94 f	7.4 ± 0.211 e	88.3 ± 1.12 f
	90	86.3 ± 1.19 c	54.5 ± 0.82 c	22.3 ± 1.01 c	101.6 ± 0.87 c	45.3 ± 1.52 c	13.3 ± 0.322 cd	157.7 ± 3.33 c
Zeolite + *Caulobacter* sp. MN13	0	30.7 ± 0.15 g	17.3 ± 0.16 g	5.8 ± 0.09 g	34.5 ± 0.21 f	13.5 ± 0.06 g	3.6 ± 0.021 f	56.7 ± 1.63 g
	90	67.0 ± 0.38 de	41.5 ± 0.71 de	16.2 ± 0.4 de	75.4 ± 1.17 d	34.7 ± 0.5 de	10.1 ± 0.175 e	124.0 ± 3.74 de

Values sharing the same letter(s) in a column are statistically non-significant with each other at *p* < 0.05; the values are mean ± standard error (*n* = 4); RG: reduced glutathione; GPX: glutathione peroxidase; GR: glutathione reductase; GST: glutathione S transferase; APX: ascorbate peroxidase; CAT: catalase, and SOD: superoxide dismutase.

**Table 5:** Effect of *Caulobacter* sp. MN13 and zeolite on biochemical parameters of sesame in normal and Ni contaminated soils.

Treatments	Ni (mg kg^−1^)	Ash (%)	Crude Fibre (%)	Protein (%)	Fats (%)
Control	0	3.81 ± 0.28 bc	10.29 ± 0.37 a–c	27.28 ± 1.11 bc	14.40 ± 0.31 bc
	90	2.91 ± 0.19 d	9.23 ± 0.34 c	25.11 ± 0.84 e	11.91 ± 0.17 c
Zeolite	0	3.90 ± 0.27 bc	10.77 ± 0.39 a–c	29.88 ± 1.15 ab	16.30 ± 0.44 ab
	90	3.71 ± 0.25 c	9.85 ± 0.26 bc	25.99 ± 0.85 de	13.87 ± 0.36 bc
*Caulobacter* sp. MN13	0	4.42 ± 0.41 ab	11.89 ± 0.31 ab	29.11 ± 1.14 ab	16.81 ± 0.15 ab
	90	3.91 ± 0.27 bc	10.17 ± 0.29 bc	26.14 ± 1.03 cd	13.94 ± 0.18 bc
Zeolite + *Caulobacter* sp. MN13	0	4.63 ± 0.43 a	12.23 ± 0.41 a	31.99 ± 1.11 a	18.91 ± 0.12 a
	90	4.11 ± 0.4 a–c	11.49 ± 0.34 ab	30.88 ± 0.94 ab	14.11 ± 0.23 bc

Values sharing the same letter(s) in a column are statistically non-significant with each other at *p* < 0.05; the values are mean ± standard error (*n* = 4).

## Supplementary Materials

The following are available online at https://www.mdpi.com/1660-4601/17/23/8859/s1, Table S1. Type of growth of *Caulobacter* sp. MN13 at different concentrations of nickel (Ni).
