# Supplementary material for: Mitigation of Nickel Toxicity and Growth Promotion in Sesame through the Application of a Bacterial Endophyte and Zeolite in Nickel Contaminated Soil
Source: Int J Environ Res Public Health. 2020 Nov 28;17(23):8859. doi: 10.3390/ijerph17238859 (PMC7730600; doi:10.3390/ijerph17238859)
Supplement: Supplementary file 1 [file ijerph-17-08859-s001.pdf]

International Journal of
Environmental Research and Public Health

Table S1. Type of Growth of *Caulobacter* sp. MN13 at different Concentrations of Nickel (Ni).

Ni Concentration as $NiSO_4$ ($\mu g\ mL^{-1}$)	Type of Growth on Petri-Plates	Bacterial Growth in Broth (OD_{600})
0	Very heavy growth	1.14 ± 0.04
30	Heavy growth	1.07 ± 0.04
60	Medium growth	0.80 ± 0.03
90	Light growth	0.52 ± 0.02
120	Very light growth	0.33 ± 0.03
160	No growth	0.10 ± 0.02

The values are mean ± standard error ($n = 4$).

© 2020 by the authors. Licensee MDPI, Basel, Switzerland. This article is an open access article distributed under the terms and conditions of the Creative Commons Attribution (CC BY) license (http://creativecommons.org/licenses/by/4.0/).
